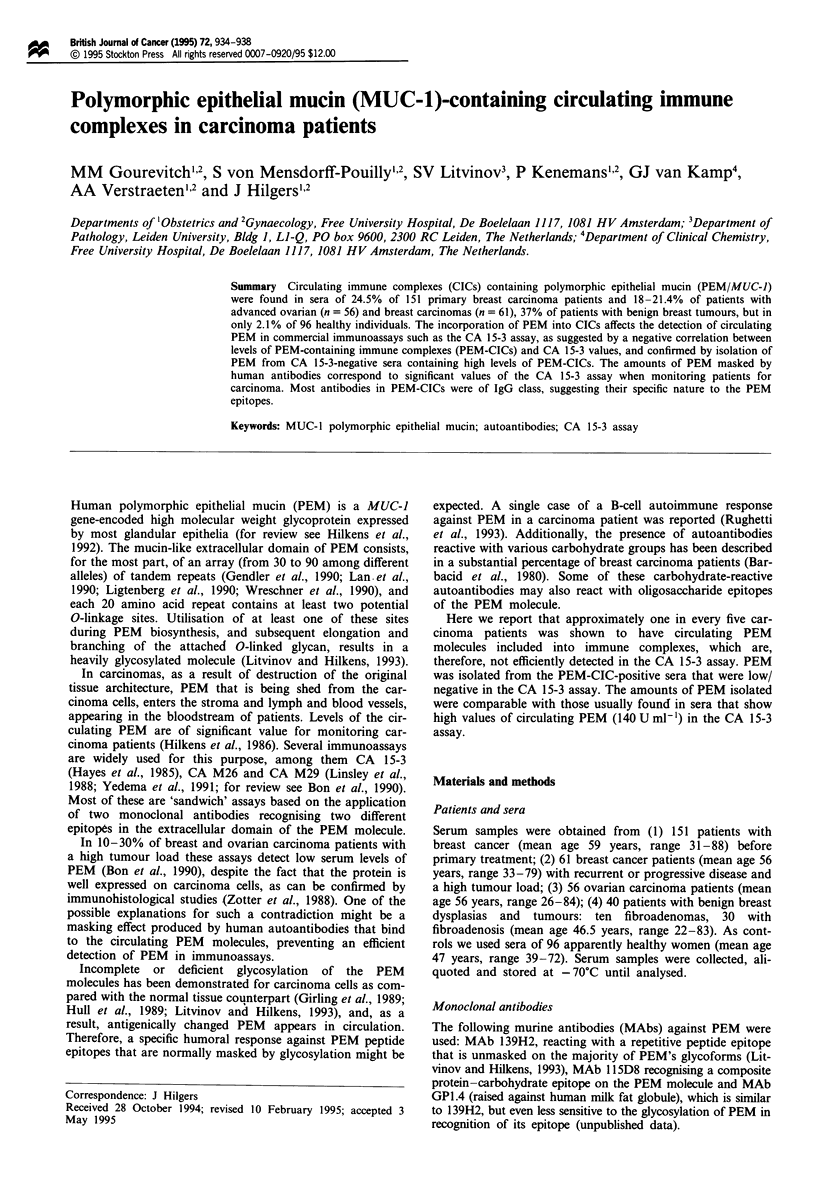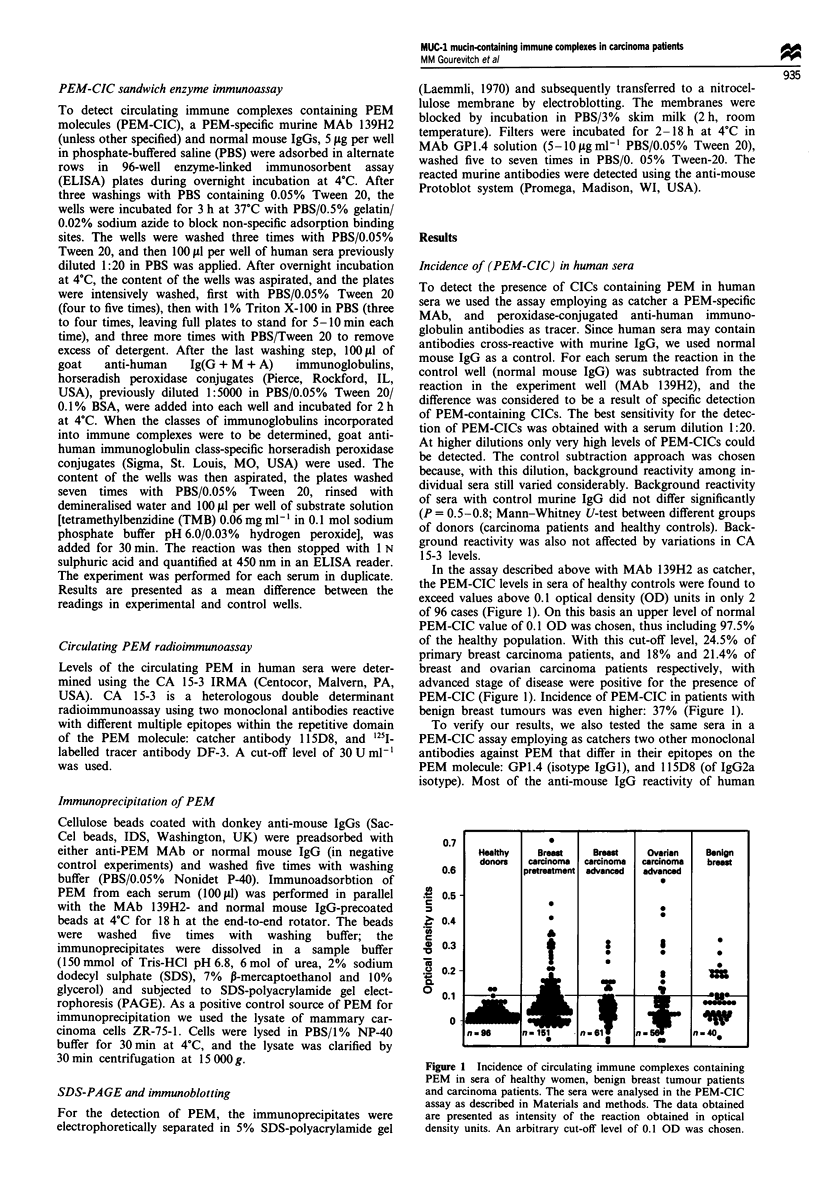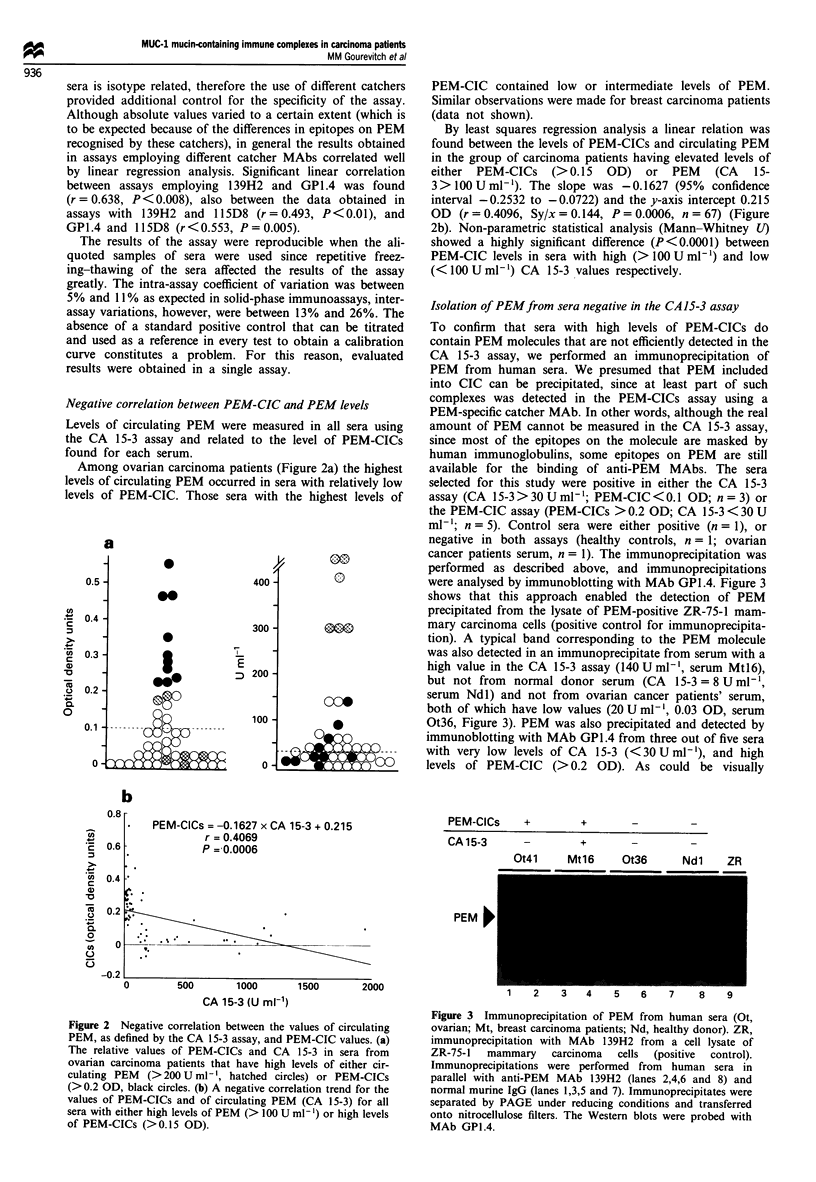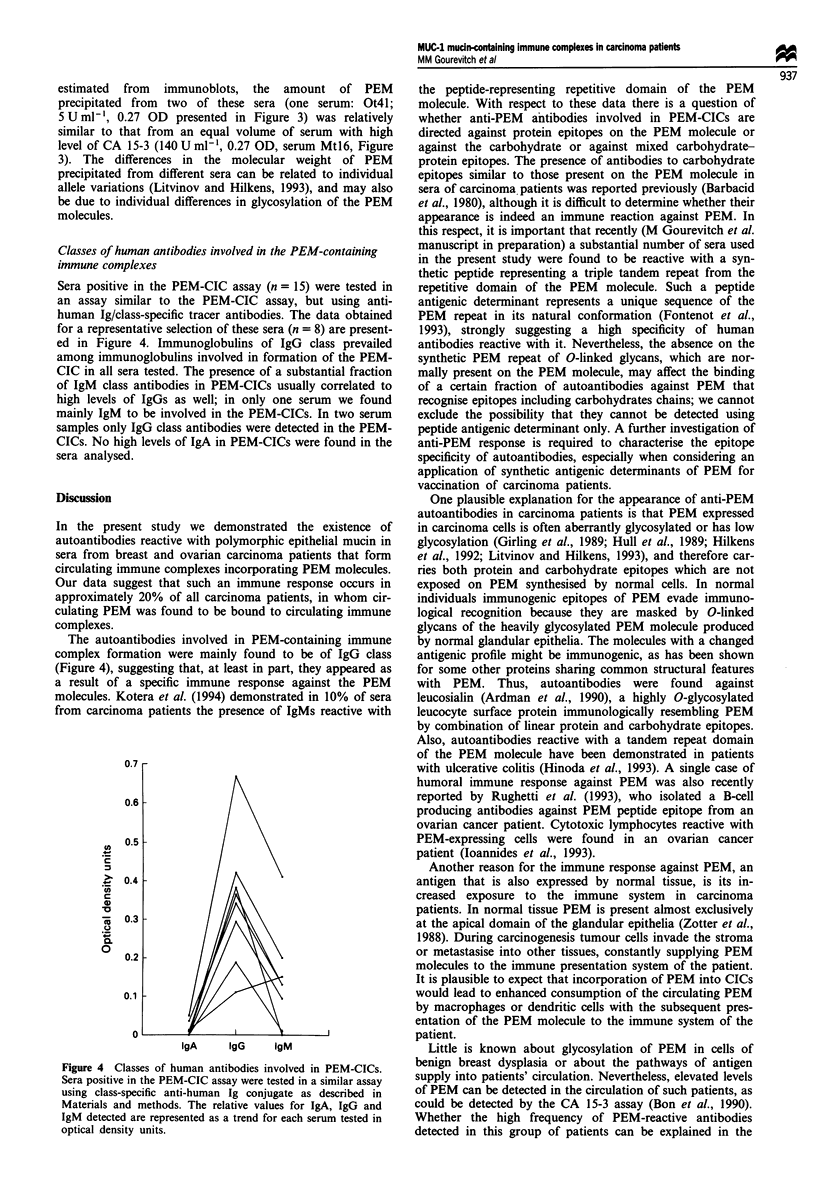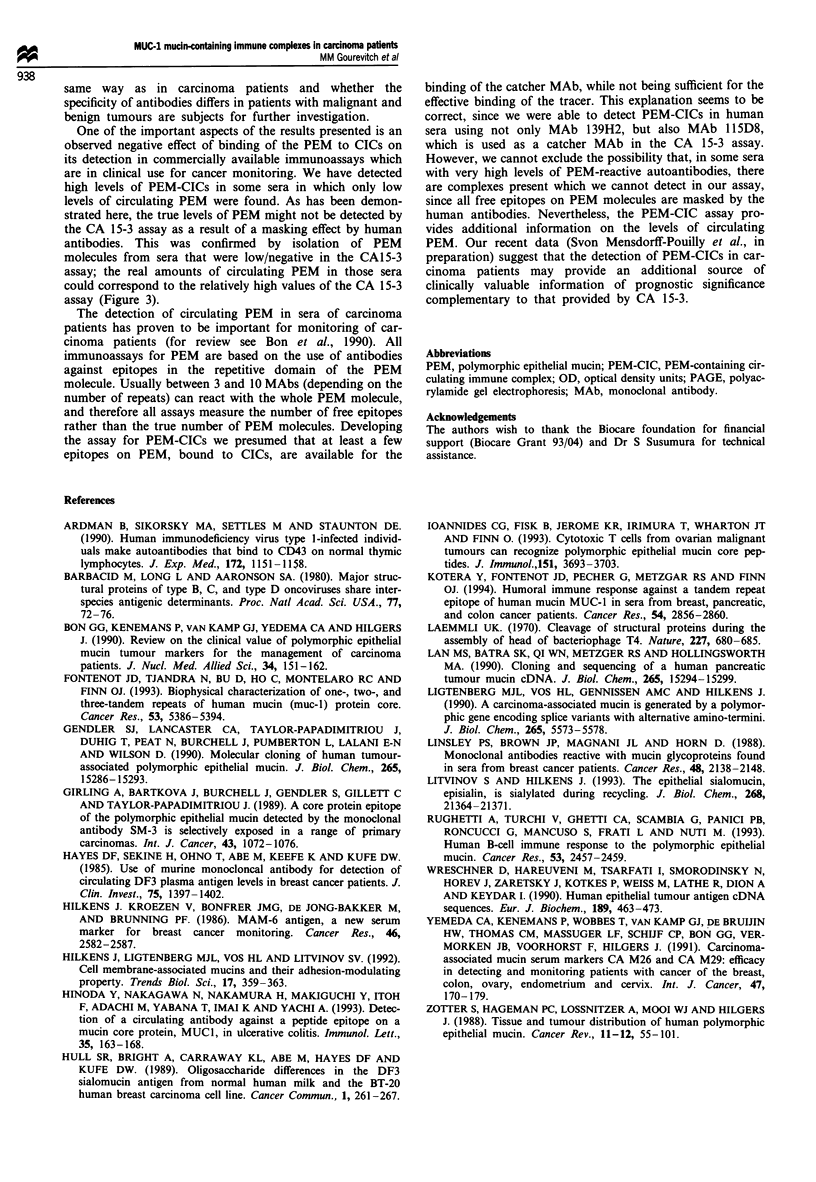# Polymorphic epithelial mucin (MUC-1)-containing circulating immune complexes in carcinoma patients.

**DOI:** 10.1038/bjc.1995.436

**Published:** 1995-10

**Authors:** M. M. Gourevitch, S. von Mensdorff-Pouilly, S. V. Litvinov, P. Kenemans, G. J. van Kamp, A. A. Verstraeten, J. Hilgers

**Affiliations:** Department of Obstetrics and Gynaecology, Free University Hospital, Amsterdam, The Netherlands.

## Abstract

**Images:**


British Journal of Cancer (1995) 72, 934–938
© 1995 Stockton Press All rights reserved 0007-0920/95 $12.00

### Polymorphic epithelial mucin (MUC-1)-containing circulating immune complexes in carcinoma patients

MM Gourevitch[1,2], S von Mensdorff-Pouilly[1,2], SV Litvinov[3], P Kenemans[1,2], GJ van Kamp[4], AA Verstraeten[1,2] and J Hilgers[1,2]

*Departments of [1]Obstetrics and [2]Gynaecology, Free University Hospital, De Boelelaan 1117, 1081 HV Amsterdam; [3]Department of Pathology, Leiden University, Bldg 1, L1-Q, PO box 9600, 2300 RC Leiden, The Netherlands; [4]Department of Clinical Chemistry, Free University Hospital, De Boelelaan 1117, 1081 HV Amsterdam, The Netherlands.*

**Summary** Circulating immune complexes (CICs) containing polymorphic epithelial mucin (PEM/*MUC-1*) were found in sera of 24.5% of 151 primary breast carcinoma patients and 18–21.4% of patients with advanced ovarian ($n = 56$) and breast carcinomas ($n = 61$), 37% of patients with benign breast tumours, but in only 2.1% of 96 healthy individuals. The incorporation of PEM into CICs affects the detection of circulating PEM in commercial immunoassays such as the CA 15-3 assay, as suggested by a negative correlation between levels of PEM-containing immune complexes (PEM-CICs) and CA 15-3 values, and confirmed by isolation of PEM from CA 15-3-negative sera containing high levels of PEM-CICs. The amounts of PEM masked by human antibodies correspond to significant values of the CA 15-3 assay when monitoring patients for carcinoma. Most antibodies in PEM-CICs were of IgG class, suggesting their specific nature to the PEM epitopes.

**Keywords:** MUC-1 polymorphic epithelial mucin; autoantibodies; CA 15-3 assay

Human polymorphic epithelial mucin (PEM) is a *MUC-1* gene-encoded high molecular weight glycoprotein expressed by most glandular epithelia (for review see Hilkens *et al.*, 1992). The mucin-like extracellular domain of PEM consists, for the most part, of an array (from 30 to 90 among different alleles) of tandem repeats (Gendler *et al.*, 1990; Lan *et al.*, 1990; Ligtenberg *et al.*, 1990; Wreschner *et al.*, 1990), and each 20 amino acid repeat contains at least two potential *O*-linkage sites. Utilisation of at least one of these sites during PEM biosynthesis, and subsequent elongation and branching of the attached *O*-linked glycan, results in a heavily glycosylated molecule (Litvinov and Hilkens, 1993).

In carcinomas, as a result of destruction of the original tissue architecture, PEM that is being shed from the carcinoma cells, enters the stroma and lymph and blood vessels, appearing in the bloodstream of patients. Levels of the circulating PEM are of significant value for monitoring carcinoma patients (Hilkens *et al.*, 1986). Several immunoassays are widely used for this purpose, among them CA 15-3 (Hayes *et al.*, 1985), CA M26 and CA M29 (Linsley *et al.*, 1988; Yedema *et al.*, 1991; for review see Bon *et al.*, 1990). Most of these are 'sandwich' assays based on the application of two monoclonal antibodies recognising two different epitopes in the extracellular domain of the PEM molecule.

In 10–30% of breast and ovarian carcinoma patients with a high tumour load these assays detect low serum levels of PEM (Bon *et al.*, 1990), despite the fact that the protein is well expressed on carcinoma cells, as can be confirmed by immunohistological studies (Zotter *et al.*, 1988). One of the possible explanations for such a contradiction might be a masking effect produced by human autoantibodies that bind to the circulating PEM molecules, preventing an efficient detection of PEM in immunoassays.

Incomplete or deficient glycosylation of the PEM molecules has been demonstrated for carcinoma cells as compared with the normal tissue counterpart (Girling *et al.*, 1989; Hull *et al.*, 1989; Litvinov and Hilkens, 1993), and, as a result, antigenically changed PEM appears in circulation. Therefore, a specific humoral response against PEM peptide epitopes that are normally masked by glycosylation might be expected. A single case of a B-cell autoimmune response against PEM in a carcinoma patient was reported (Rughetti *et al.*, 1993). Additionally, the presence of autoantibodies reactive with various carbohydrate groups has been described in a substantial percentage of breast carcinoma patients (Barbacid *et al.*, 1980). Some of these carbohydrate-reactive autoantibodies may also react with oligosaccharide epitopes of the PEM molecule.

Here we report that approximately one in every five carcinoma patients was shown to have circulating PEM molecules included into immune complexes, which are, therefore, not efficiently detected in the CA 15-3 assay. PEM was isolated from the PEM-CIC-positive sera that were low/negative in the CA 15-3 assay. The amounts of PEM isolated were comparable with those usually found in sera that show high values of circulating PEM (140 U $ml^{-1}$) in the CA 15-3 assay.

Correspondence: J Hilgers
Received 28 October 1994; revised 10 February 1995; accepted 3 May 1995

### Materials and methods

### *Patients and sera*

Serum samples were obtained from (1) 151 patients with breast cancer (mean age 59 years, range 31–88) before primary treatment; (2) 61 breast cancer patients (mean age 56 years, range 33–79) with recurrent or progressive disease and a high tumour load; (3) 56 ovarian carcinoma patients (mean age 56 years, range 26–84); (4) 40 patients with benign breast dysplasias and tumours: ten fibroadenomas, 30 with fibroadenosis (mean age 46.5 years, range 22–83). As controls we used sera of 96 apparently healthy women (mean age 47 years, range 39–72). Serum samples were collected, aliquoted and stored at −70°C until analysed.

### *Monoclonal antibodies*

The following murine antibodies (MAbs) against PEM were used: MAb 139H2, reacting with a repetitive peptide epitope that is unmasked on the majority of PEM's glycoforms (Litvinov and Hilkens, 1993), MAb 115D8 recognising a composite protein–carbohydrate epitope on the PEM molecule and MAb GP1.4 (raised against human milk fat globule), which is similar to 139H2, but even less sensitive to the glycosylation of PEM in recognition of its epitope (unpublished data).

*PEM-CIC sandwich enzyme immunoassay*

To detect circulating immune complexes containing PEM molecules (PEM-CIC), a PEM-specific murine MAb 139H2 (unless other specified) and normal mouse IgGs, 5 μg per well in phosphate-buffered saline (PBS) were adsorbed in alternate rows in 96-well enzyme-linked immunosorbent assay (ELISA) plates during overnight incubation at 4°C. After three washings with PBS containing 0.05% Tween 20, the wells were incubated for 3 h at 37°C with PBS/0.5% gelatin/0.02% sodium azide to block non-specific adsorption binding sites. The wells were washed three times with PBS/0.05% Tween 20, and then 100 μl per well of human sera previously diluted 1:20 in PBS was applied. After overnight incubation at 4°C, the content of the wells was aspirated, and the plates were intensively washed, first with PBS/0.05% Tween 20 (four to five times), then with 1% Triton X-100 in PBS (three to four times, leaving full plates to stand for 5–10 min each time), and three more times with PBS/Tween 20 to remove excess of detergent. After the last washing step, 100 μl of goat anti-human Ig(G + M + A) immunoglobulins, horseradish peroxidase conjugates (Pierce, Rockford, IL, USA), previously diluted 1:5000 in PBS/0.05% Tween 20/0.1% BSA, were added into each well and incubated for 2 h at 4°C. When the classes of immunoglobulins incorporated into immune complexes were to be determined, goat anti-human immunoglobulin class-specific horseradish peroxidase conjugates (Sigma, St. Louis, MO, USA) were used. The content of the wells was then aspirated, the plates washed seven times with PBS/0.05% Tween 20, rinsed with demineralised water and 100 μl per well of substrate solution [tetramethylbenzidine (TMB) 0.06 mg $ml^{-1}$ in 0.1 mol sodium phosphate buffer pH 6.0/0.03% hydrogen peroxide], was added for 30 min. The reaction was then stopped with 1 N sulphuric acid and quantified at 450 nm in an ELISA reader. The experiment was performed for each serum in duplicate. Results are presented as a mean difference between the readings in experimental and control wells.

*Circulating PEM radioimmunoassay*

Levels of the circulating PEM in human sera were determined using the CA 15-3 IRMA (Centocor, Malvern, PA, USA). CA 15-3 is a heterologous double determinant radioimmunoassay using two monoclonal antibodies reactive with different multiple epitopes within the repetitive domain of the PEM molecule: catcher antibody 115D8, and $^{125}$I-labelled tracer antibody DF-3. A cut-off level of 30 U $ml^{-1}$ was used.

*Immunoprecipitation of PEM*

Cellulose beads coated with donkey anti-mouse IgGs (Sac-Cel beads, IDS, Washington, UK) were preadsorbed with either anti-PEM MAb or normal mouse IgG (in negative control experiments) and washed five times with washing buffer (PBS/0.05% Nonidet P-40). Immunoadsorbtion of PEM from each serum (100 μl) was performed in parallel with the MAb 139H2- and normal mouse IgG-precoated beads at 4°C for 18 h at the end-to-end rotator. The beads were washed five times with washing buffer; the immunoprecipitates were dissolved in a sample buffer (150 mmol of Tris-HCl pH 6.8, 6 mol of urea, 2% sodium dodecyl sulphate (SDS), 7% β-mercaptoethanol and 10% glycerol) and subjected to SDS-polyacrylamide gel electrophoresis (PAGE). As a positive control source of PEM for immunoprecipitation we used the lysate of mammary carcinoma cells ZR-75-1. Cells were lysed in PBS/1% NP-40 buffer for 30 min at 4°C, and the lysate was clarified by 30 min centrifugation at 15 000 *g*.

*SDS-PAGE and immunoblotting*

For the detection of PEM, the immunoprecipitates were electrophoretically separated in 5% SDS-polyacrylamide gel (Laemmli, 1970) and subsequently transferred to a nitrocellulose membrane by electroblotting. The membranes were blocked by incubation in PBS/3% skim milk (2 h, room temperature). Filters were incubated for 2–18 h at 4°C in MAb GP1.4 solution (5–10 μg $ml^{-1}$ PBS/0.05% Tween 20), washed five to seven times in PBS/0. 05% Tween-20. The reacted murine antibodies were detected using the anti-mouse Protoblot system (Promega, Madison, WI, USA).

### Results

*Incidence of (PEM-CIC) in human sera*

To detect the presence of CICs containing PEM in human sera we used the assay employing as catcher a PEM-specific MAb, and peroxidase-conjugated anti-human immunoglobulin antibodies as tracer. Since human sera may contain antibodies cross-reactive with murine IgG, we used normal mouse IgG as a control. For each serum the reaction in the control well (normal mouse IgG) was subtracted from the reaction in the experiment well (MAb 139H2), and the difference was considered to be a result of specific detection of PEM-containing CICs. The best sensitivity for the detection of PEM-CICs was obtained with a serum dilution 1:20. At higher dilutions only very high levels of PEM-CICs could be detected. The control subtraction approach was chosen because, with this dilution, background reactivity among individual sera still varied considerably. Background reactivity of sera with control murine IgG did not differ significantly ($P = 0.5–0.8$; Mann–Whitney *U*-test between different groups of donors (carcinoma patients and healthy controls). Background reactivity was also not affected by variations in CA 15-3 levels.

In the assay described above with MAb 139H2 as catcher, the PEM-CIC levels in sera of healthy controls were found to exceed values above 0.1 optical density (OD) units in only 2 of 96 cases (Figure 1). On this basis an upper level of normal PEM-CIC value of 0.1 OD was chosen, thus including 97.5% of the healthy population. With this cut-off level, 24.5% of primary breast carcinoma patients, and 18% and 21.4% of breast and ovarian carcinoma patients respectively, with advanced stage of disease were positive for the presence of PEM-CIC (Figure 1). Incidence of PEM-CIC in patients with benign breast tumours was even higher: 37% (Figure 1).

To verify our results, we also tested the same sera in a PEM-CIC assay employing as catchers two other monoclonal antibodies against PEM that differ in their epitopes on the PEM molecule: GP1.4 (isotype IgG1), and 115D8 (of IgG2a isotype). Most of the anti-mouse IgG reactivity of human

**Figure 1** Incidence of circulating immune complexes containing PEM in sera of healthy women, benign breast tumour patients and carcinoma patients. The sera were analysed in the PEM-CIC assay as described in Materials and methods. The data obtained are presented as intensity of the reaction obtained in optical density units. An arbitrary cut-off level of 0.1 OD was chosen.

sera is isotype related, therefore the use of different catchers provided additional control for the specificity of the assay. Although absolute values varied to a certain extent (which is to be expected because of the differences in epitopes on PEM recognised by these catchers), in general the results obtained in assays employing different catcher MAbs correlated well by linear regression analysis. Significant linear correlation between assays employing 139H2 and GP1.4 was found ($r = 0.638$, $P < 0.008$), also between the data obtained in assays with 139H2 and 115D8 ($r = 0.493$, $P < 0.01$), and GP1.4 and 115D8 ($r < 0.553$, $P = 0.005$).

The results of the assay were reproducible when the aliquoted samples of sera were used since repetitive freezing–thawing of the sera affected the results of the assay greatly. The intra-assay coefficient of variation was between 5% and 11% as expected in solid-phase immunoassays, inter-assay variations, however, were between 13% and 26%. The absence of a standard positive control that can be titrated and used as a reference in every test to obtain a calibration curve constitutes a problem. For this reason, evaluated results were obtained in a single assay.

### *Negative correlation between PEM-CIC and PEM levels*

Levels of circulating PEM were measured in all sera using the CA 15-3 assay and related to the level of PEM-CICs found for each serum.

Among ovarian carcinoma patients (Figure 2a) the highest levels of circulating PEM occurred in sera with relatively low levels of PEM-CIC. Those sera with the highest levels of PEM-CIC contained low or intermediate levels of PEM. Similar observations were made for breast carcinoma patients (data not shown).

By least squares regression analysis a linear relation was found between the levels of PEM-CICs and circulating PEM in the group of carcinoma patients having elevated levels of either PEM-CICs (>0.15 OD) or PEM (CA 15-3 > 100 U ml$^{-1}$). The slope was $-0.1627$ (95% confidence interval $-0.2532$ to $-0.0722$) and the $y$-axis intercept 0.215 OD ($r = 0.4096$, $Sy/x = 0.144$, $P = 0.0006$, $n = 67$) (Figure 2b). Non-parametric statistical analysis (Mann–Whitney $U$) showed a highly significant difference ($P < 0.0001$) between PEM-CIC levels in sera with high (> 100 U ml$^{-1}$) and low (< 100 U ml$^{-1}$) CA 15-3 values respectively.

### *Isolation of PEM from sera negative in the CA15-3 assay*

To confirm that sera with high levels of PEM-CICs do contain PEM molecules that are not efficiently detected in the CA 15-3 assay, we performed an immunoprecipitation of PEM from human sera. We presumed that PEM included into CIC can be precipitated, since at least part of such complexes was detected in the PEM-CICs assay using a PEM-specific catcher MAb. In other words, although the real amount of PEM cannot be measured in the CA 15-3 assay, since most of the epitopes on the molecule are masked by human immunoglobulins, some epitopes on PEM are still available for the binding of anti-PEM MAbs. The sera selected for this study were positive in either the CA 15-3 assay (CA 15-3 > 30 U ml$^{-1}$; PEM-CIC < 0.1 OD; $n = 3$) or the PEM-CIC assay (PEM-CICs > 0.2 OD; CA 15-3 < 30 U ml$^{-1}$; $n = 5$). Control sera were either positive ($n = 1$), or negative in both assays (healthy controls, $n = 1$; ovarian cancer patients serum, $n = 1$). The immunoprecipitation was performed as described above, and immunoprecipitations were analysed by immunoblotting with MAb GP1.4. Figure 3 shows that this approach enabled the detection of PEM precipitated from the lysate of PEM-positive ZR-75-1 mammary carcinoma cells (positive control for immunoprecipitation). A typical band corresponding to the PEM molecule was also detected in an immunoprecipitate from serum with a high value in the CA 15-3 assay (140 U ml$^{-1}$, serum Mt16), but not from normal donor serum (CA 15-3 = 8 U ml$^{-1}$, serum Nd1) and not from ovarian cancer patients' serum, both of which have low values (20 U ml$^{-1}$, 0.03 OD, serum Ot36, Figure 3). PEM was also precipitated and detected by immunoblotting with MAb GP1.4 from three out of five sera with very low levels of CA 15-3 (< 30 U ml$^{-1}$), and high levels of PEM-CIC (> 0.2 OD). As could be visually

**Figure 2** Negative correlation between the values of circulating PEM, as defined by the CA 15-3 assay, and PEM-CIC values. (**a**) The relative values of PEM-CICs and CA 15-3 in sera from ovarian carcinoma patients that have high levels of either circulating PEM (> 200 U ml$^{-1}$, hatched circles) or PEM-CICs (> 0.2 OD, black circles). (**b**) A negative correlation trend for the values of PEM-CICs and of circulating PEM (CA 15-3) for all sera with either high levels of PEM (> 100 U ml$^{-1}$) or high levels of PEM-CICs (> 0.15 OD).

**Figure 3** Immunoprecipitation of PEM from human sera (Ot, ovarian; Mt, breast carcinoma patients; Nd, healthy donor). ZR, immunoprecipitation with MAb 139H2 from a cell lysate of ZR-75-1 mammary carcinoma cells (positive control). Immunoprecipitations were performed from human sera in parallel with anti-PEM MAb 139H2 (lanes 2,4,6 and 8) and normal murine IgG (lanes 1,3,5 and 7). Immunoprecipitates were separated by PAGE under reducing conditions and transferred onto nitrocellulose filters. The Western blots were probed with MAb GP1.4.

estimated from immunoblots, the amount of PEM precipitated from two of these sera (one serum: Ot41; 5 U $ml^{-1}$, 0.27 OD presented in Figure 3) was relatively similar to that from an equal volume of serum with high level of CA 15-3 (140 U $ml^{-1}$, 0.27 OD, serum Mt16, Figure 3). The differences in the molecular weight of PEM precipitated from different sera can be related to individual allele variations (Litvinov and Hilkens, 1993), and may also be due to individual differences in glycosylation of the PEM molecules.

### *Classes of human antibodies involved in the PEM-containing immune complexes*

Sera positive in the PEM-CIC assay ($n = 15$) were tested in an assay similar to the PEM-CIC assay, but using anti-human Ig/class-specific tracer antibodies. The data obtained for a representative selection of these sera ($n = 8$) are presented in Figure 4. Immunoglobulins of IgG class prevailed among immunoglobulins involved in formation of the PEM-CIC in all sera tested. The presence of a substantial fraction of IgM class antibodies in PEM-CICs usually correlated to high levels of IgGs as well; in only one serum we found mainly IgM to be involved in the PEM-CICs. In two serum samples only IgG class antibodies were detected in the PEM-CICs. No high levels of IgA in PEM-CICs were found in the sera analysed.

### Discussion

In the present study we demonstrated the existence of autoantibodies reactive with polymorphic epithelial mucin in sera from breast and ovarian carcinoma patients that form circulating immune complexes incorporating PEM molecules. Our data suggest that such an immune response occurs in approximately 20% of all carcinoma patients, in whom circulating PEM was found to be bound to circulating immune complexes.

The autoantibodies involved in PEM-containing immune complex formation were mainly found to be of IgG class (Figure 4), suggesting that, at least in part, they appeared as a result of a specific immune response against the PEM molecules. Kotera *et al.* (1994) demonstrated in 10% of sera from carcinoma patients the presence of IgMs reactive with the peptide-representing repetitive domain of the PEM molecule. With respect to these data there is a question of whether anti-PEM antibodies involved in PEM-CICs are directed against protein epitopes on the PEM molecule or against the carbohydrate or against mixed carbohydrate–protein epitopes. The presence of antibodies to carbohydrate epitopes similar to those present on the PEM molecule in sera of carcinoma patients was reported previously (Barbacid *et al.*, 1980), although it is difficult to determine whether their appearance is indeed an immune reaction against PEM. In this respect, it is important that recently (M Gourevitch *et al.* manuscript in preparation) a substantial number of sera used in the present study were found to be reactive with a synthetic peptide representing a triple tandem repeat from the repetitive domain of the PEM molecule. Such a peptide antigenic determinant represents a unique sequence of the PEM repeat in its natural conformation (Fontenot *et al.*, 1993), strongly suggesting a high specificity of human antibodies reactive with it. Nevertheless, the absence on the synthetic PEM repeat of *O*-linked glycans, which are normally present on the PEM molecule, may affect the binding of a certain fraction of autoantibodies against PEM that recognise epitopes including carbohydrates chains; we cannot exclude the possibility that they cannot be detected using peptide antigenic determinant only. A further investigation of anti-PEM response is required to characterise the epitope specificity of autoantibodies, especially when considering an application of synthetic antigenic determinants of PEM for vaccination of carcinoma patients.

**Figure 4** Classes of human antibodies involved in PEM-CICs. Sera positive in the PEM-CIC assay were tested in a similar assay using class-specific anti-human Ig conjugate as described in Materials and methods. The relative values for IgA, IgG and IgM detected are represented as a trend for each serum tested in optical density units.

One plausible explanation for the appearance of anti-PEM autoantibodies in carcinoma patients is that PEM expressed in carcinoma cells is often aberrantly glycosylated or has low glycosylation (Girling *et al.*, 1989; Hull *et al.*, 1989; Hilkens *et al.*, 1992; Litvinov and Hilkens, 1993), and therefore carries both protein and carbohydrate epitopes which are not exposed on PEM synthesised by normal cells. In normal individuals immunogenic epitopes of PEM evade immunological recognition because they are masked by *O*-linked glycans of the heavily glycosylated PEM molecule produced by normal glandular epithelia. The molecules with a changed antigenic profile might be immunogenic, as has been shown for some other proteins sharing common structural features with PEM. Thus, autoantibodies were found against leucosialin (Ardman *et al.*, 1990), a highly *O*-glycosylated leucocyte surface protein immunologically resembling PEM by combination of linear protein and carbohydrate epitopes. Also, autoantibodies reactive with a tandem repeat domain of the PEM molecule have been demonstrated in patients with ulcerative colitis (Hinoda *et al.*, 1993). A single case of humoral immune response against PEM was also recently reported by Rughetti *et al.* (1993), who isolated a B-cell producing antibodies against PEM peptide epitope from an ovarian cancer patient. Cytotoxic lymphocytes reactive with PEM-expressing cells were found in an ovarian cancer patient (Ioannides *et al.*, 1993).

Another reason for the immune response against PEM, an antigen that is also expressed by normal tissue, is its increased exposure to the immune system in carcinoma patients. In normal tissue PEM is present almost exclusively at the apical domain of the glandular epithelia (Zotter *et al.*, 1988). During carcinogenesis tumour cells invade the stroma or metastasise into other tissues, constantly supplying PEM molecules to the immune presentation system of the patient. It is plausible to expect that incorporation of PEM into CICs would lead to enhanced consumption of the circulating PEM by macrophages or dendritic cells with the subsequent presentation of the PEM molecule to the immune system of the patient.

Little is known about glycosylation of PEM in cells of benign breast dysplasia or about the pathways of antigen supply into patients' circulation. Nevertheless, elevated levels of PEM can be detected in the circulation of such patients, as could be detected by the CA 15-3 assay (Bon *et al.*, 1990). Whether the high frequency of PEM-reactive antibodies detected in this group of patients can be explained in the same way as in carcinoma patients and whether the specificity of antibodies differs in patients with malignant and benign tumours are subjects for further investigation.

One of the important aspects of the results presented is an observed negative effect of binding of the PEM to CICs on its detection in commercially available immunoassays which are in clinical use for cancer monitoring. We have detected high levels of PEM-CICs in some sera in which only low levels of circulating PEM were found. As has been demonstrated here, the true levels of PEM might not be detected by the CA 15-3 assay as a result of a masking effect by human antibodies. This was confirmed by isolation of PEM molecules from sera that were low/negative in the CA15-3 assay; the real amounts of circulating PEM in those sera could correspond to the relatively high values of the CA 15-3 assay (Figure 3).

The detection of circulating PEM in sera of carcinoma patients has proven to be important for monitoring of carcinoma patients (for review see Bon *et al.*, 1990). All immunoassays for PEM are based on the use of antibodies against epitopes in the repetitive domain of the PEM molecule. Usually between 3 and 10 MAbs (depending on the number of repeats) can react with the whole PEM molecule, and therefore all assays measure the number of free epitopes rather than the true number of PEM molecules. Developing the assay for PEM-CICs we presumed that at least a few epitopes on PEM, bound to CICs, are available for the binding of the catcher MAb, while not being sufficient for the effective binding of the tracer. This explanation seems to be correct, since we were able to detect PEM-CICs in human sera using not only MAb 139H2, but also MAb 115D8, which is used as a catcher MAb in the CA 15-3 assay. However, we cannot exclude the possibility that, in some sera with very high levels of PEM-reactive autoantibodies, there are complexes present which we cannot detect in our assay, since all free epitopes on PEM molecules are masked by the human antibodies. Nevertheless, the PEM-CIC assay provides additional information on the levels of circulating PEM. Our recent data (Svon Mensdorff-Pouilly *et al.*, in preparation) suggest that the detection of PEM-CICs in carcinoma patients may provide an additional source of clinically valuable information of prognostic significance complementary to that provided by CA 15-3.

### Abbreviations

PEM, polymorphic epithelial mucin; PEM-CIC, PEM-containing circulating immune complex; OD, optical density units; PAGE, polyacrylamide gel electrophoresis; MAb, monoclonal antibody.

### Acknowledgements

The authors wish to thank the Biocare foundation for financial support (Biocare Grant 93/04) and Dr S Susumura for technical assistance.

### References

ARDMAN B, SIKORSKY MA, SETTLES M AND STAUNTON DE. (1990). Human immunodeficiency virus type 1-infected individuals make autoantibodies that bind to CD43 on normal thymic lymphocytes. *J. Exp. Med.*, **172**, 1151–1158.

BARBACID M, LONG L AND AARONSON SA. (1980). Major structural proteins of type B, C, and type D oncoviruses share interspecies antigenic determinants. *Proc. Natl Acad. Sci. USA.*, **77**, 72–76.

BON GG, KENEMANS P, VAN KAMP GJ, YEDEMA CA AND HILGERS J. (1990). Review on the clinical value of polymorphic epithelial mucin tumour markers for the management of carcinoma patients. *J. Nucl. Med. Allied Sci.*, **34**, 151–162.

FONTENOT JD, TJANDRA N, BU D, HO C, MONTELARO RC AND FINN OJ. (1993). Biophysical characterization of one-, two-, and three-tandem repeats of human mucin (muc-1) protein core. *Cancer Res.*, **53**, 5386–5394.

GENDLER SJ, LANCASTER CA, TAYLOR-PAPADIMITRIOU J, DUHIG T, PEAT N, BURCHELL J, PUMBERTON L, LALANI E-N AND WILSON D. (1990). Molecular cloning of human tumour-associated polymorphic epithelial mucin. *J. Biol. Chem.*, **265**, 15286–15293.

GIRLING A, BARTKOVA J, BURCHELL J, GENDLER S, GILLETT C AND TAYLOR-PAPADIMITRIOU J. (1989). A core protein epitope of the polymorphic epithelial mucin detected by the monoclonal antibody SM-3 is selectively exposed in a range of primary carcinomas. *Int. J. Cancer*, **43**, 1072–1076.

HAYES DF, SEKINE H, OHNO T, ABE M, KEEFE K AND KUFE DW. (1985). Use of murine monocloncal antibody for detection of circulating DF3 plasma antigen levels in breast cancer patients. *J. Clin. Invest.*, **75**, 1397–1402.

HILKENS J. KROEZEN V, BONFRER JMG, DE JONG-BAKKER M, AND BRUNNING PF. (1986). MAM-6 antigen, a new serum marker for breast cancer monitoring. *Cancer Res.*, **46**, 2582–2587.

HILKENS J, LIGTENBERG MJL, VOS HL AND LITVINOV SV. (1992). Cell membrane-associated mucins and their adhesion-modulating property. *Trends Biol. Sci.*, **17**, 359–363.

HINODA Y, NAKAGAWA N, NAKAMURA H, MAKIGUCHI Y, ITOH F, ADACHI M, YABANA T, IMAI K AND YACHI A. (1993). Detection of a circulating antibody against a peptide epitope on a mucin core protein, MUC1, in ulcerative colitis. *Immunol. Lett.*, **35**, 163–168.

HULL SR, BRIGHT A, CARRAWAY KL, ABE M, HAYES DF AND KUFE DW. (1989). Oligosaccharide differences in the DF3 sialomucin antigen from normal human milk and the BT-20 human breast carcinoma cell line. *Cancer Commun.*, **1**, 261–267.

IOANNIDES CG, FISK B, JEROME KR, IRIMURA T, WHARTON JT AND FINN O. (1993). Cytotoxic T cells from ovarian malignant tumours can recognize polymorphic epithelial mucin core peptides. *J. Immunol.*,**151**, 3693–3703.

KOTERA Y, FONTENOT JD, PECHER G, METZGAR RS AND FINN OJ. (1994). Humoral immune response against a tandem repeat epitope of human mucin MUC-1 in sera from breast, pancreatic, and colon cancer patients. *Cancer Res.*, **54**, 2856–2860.

LAEMMLI UK. (1970). Cleavage of structural proteins during the assembly of head of bacteriophage T4. *Nature*, **227**, 680–685.

LAN MS, BATRA SK, QI WN, METZGER RS AND HOLLINGSWORTH MA. (1990). Cloning and sequencing of a human pancreatic tumour mucin cDNA. *J. Biol. Chem.*, **265**, 15294–15299.

LIGTENBERG MJL, VOS HL, GENNISSEN AMC AND HILKENS J. (1990). A carcinoma-associated mucin is generated by a polymorphic gene encoding splice variants with alternative amino-termini. *J. Biol. Chem.*, **265**, 5573–5578.

LINSLEY PS, BROWN JP, MAGNANI JL AND HORN D. (1988). Monoclonal antibodies reactive with mucin glycoproteins found in sera from breast cancer patients. *Cancer Res.*, **48**, 2138–2148.

LITVINOV S AND HILKENS J. (1993). The epithelial sialomucin, episialin, is sialylated during recycling. *J. Biol. Chem.*, **268**, 21364–21371.

RUGHETTI A, TURCHI V, GHETTI CA, SCAMBIA G, PANICI PB, RONCUCCI G, MANCUSO S, FRATI L AND NUTI M. (1993). Human B-cell immune response to the polymorphic epithelial mucin. *Cancer Res.*, **53**, 2457–2459.

WRESCHNER D, HAREUVENI M, TSARFATI I, SMORODINSKY N, HOREV J, ZARETSKY J, KOTKES P, WEISS M, LATHE R, DION A AND KEYDAR I. (1990). Human epithelial tumour antigen cDNA sequences. *Eur. J. Biochem.*, **189**, 463–473.

YEMEDA CA, KENEMANS P, WOBBES T, VAN KAMP GJ, DE BRUIJIN HW, THOMAS CM, MASSUGER LF, SCHIJF CP, BON GG, VERMORKEN JB, VOORHORST F, HILGERS J. (1991). Carcinoma-associated mucin serum markers CA M26 and CA M29: efficacy in detecting and monitoring patients with cancer of the breast, colon, ovary, endometrium and cervix. *Int. J. Cancer*, **47**, 170–179.

ZOTTER S, HAGEMAN PC, LOSSNITZER A, MOOI WJ AND HILGERS J. (1988). Tissue and tumour distribution of human polymorphic epithelial mucin. *Cancer Rev.*, **11–12**, 55–101.